# Stretched beyond our capacity: The voices of operational managers from Ekurhuleni clinics during COVID-19

**DOI:** 10.4102/curationis.v45i1.2376

**Published:** 2022-12-02

**Authors:** Siyabulela N. Wopula, Sanele E. Nene, Elizabeth Nkosi

**Affiliations:** 1 Department of Nursing, Faculty of Health Sciences, University of Johannesburg, Johannesburg, South Africa

**Keywords:** Operational manager, COVID-19 pandemic, primary healthcare, primary health care facilities, experiences

## Abstract

**Background:**

Operational Managers (OMs) in primary health care (PHC) experienced new management dynamics during the outbreak of coronavirus disease 2019 (COVID-19). They were not sufficiently prepared to deal with the extraordinary challenges brought by this global pandemic.

**Objectives:**

The aim of this study was to explore and describe the PHC OMs’ experiences of new management dynamics in PHC facilities, created by COVID-19 pandemic.

**Method:**

This study used a qualitative, exploratory, descriptive and contextual research design and a phenomenological approach. Data were collected using in-depth semi-structured individual interviews. Data saturation was reached by the 7th interview and two more interviews were done to confirm data saturation. Data analysis was conducted using Giorgi’s descriptive thematic phenomenological data analysis method. An independent coder was implored to confirm the findings. This study was guided by Rogers Diffusion of Innovation Theory. Ethical considerations were applied throughout the research process.

**Results:**

One central theme and three main themes emerged as; stretching of inadequate resources. themes; (1) budgetary cuts and increasing demands of resources, (2) insufficient of personal protective equipment, other general supplies and human resources, and (3) compromised service delivery and increased client’s dissatisfaction.

**Conclusion:**

This study revealed that OMs were over stretched and overwhelmed by the management on PHC facilities due to COVID-19 pandemic dynamics.

**Contribution:**

The findings of this study can be implemented in PHC facilities to effectively deal with future pandemics of such a nature.

## Introduction

The functions of operational managers (OMs) within a primary health care (PHC) facility are the planning, organising, implementing and monitoring of operational activities; however, the outbreak of coronavirus disease 2019 (COVID-19) disrupted this norm of OMs (Muller 2020). These managers are responsible for general operational activities and also to make certain that management processes and systems are in place and effective within PHC facilities (Jooste 2017; South African National Department of Health 2019). Operational managers need to have competence in operational management, quality improvement, information management, service delivery, resource management, infection prevention and control, leadership and cooperative governance (Madale & English 2016).

According to Jooste (2017), management dynamics are the forces that impact the management of healthcare organisations. The PHC system of South Africa categorises these management dynamics as: professional ethical consideration; resource management; common law and legislative frameworks; strategic management planning; and cooperative governance (National Department of Health Strategic Plan 2020). Munyewende (2016) went a step further and alluded that PHC facility managers deal with various health management dynamics, including increased patient flow with limited privacy, staff shortages, poor incentives and expectations of facility decongestion, highly centralised management structures and the complex disease burden that has created management dilemmas.

Coronavirus disease 2019 is defined as a severe acute respiratory syndrome characterised by fever, coughing and shortness of breath; it was first detected in Wuhan, China, in December 2019 (Lipsitch & Phil 2020). The World Health Organization (WHO) declared COVID-19 a pandemic on 11 March 2020. Within the Ekurhuleni PHC facilities, changes occurred from March 2020, when the pandemic became an uncomfortable reality in the South African healthcare facilities, influencing management dynamics. The COVID-19 pandemic posed a great threat to global health and the economy; as a result, operational and capital budgets had to be amended and reduced tremendously, resulting in comprised quality of care within PHC facilities. During the peak of the COVID-19 pandemic, it became evident and critical that OMs needed enormous support and to be equipped with resources to effectively deal with the burden that came with this unknown global pandemic (Lipsitch & Phil 2020).

Consequently, OMs had to ensure that personal protective equipment (PPE) was available, well-distributed and utilised effectively (Primary Health Care Performance Initiative 2020). Due to the ongoing and dynamic changes in the PHC environment, OMs were also tasked to continuously assess and evaluate new management dynamics through cooperative strategic management systems to achieve organisational goals and community healthcare needs (Madale & English 2016). Strategic management planning was key during this tenure to achieve sustainability in management’s capacity. These authors further stated that decentralised strategic management systems would assist facility managers in promoting health for all, eliminating OMs’ notion that they have little or no support from their immediate supervisors and are not involved in strategic planning phases.

## Problem statement

The COVID-19 pandemic exerted a heavy burden on the healthcare system, particularly on OMs, who were faced with the greatest challenges regarding the unprecedented outbreak of coronavirus worldwide. The management skills of OMs were truly tested, and this was evident by the fact that they were not sufficiently prepared to deal with the extraordinary challenges brought to the fore by this global pandemic. This resulted in role confusion, which led to the disruption of passable operational activities of PHC facilities (WHO 2020). Operational managers in these facilities were suddenly faced with this complex unknown working environment, leaving a sense of uncertainty and inadequacy in fulfilling their duties. During the time, when this pandemic was on its upwards trajectory, these OMs were expected to assume management roles and clinical roles simultaneously. They had to manage the facilities, join in the community mass screening and testing initiatives and assist in the tracing of contacts, in addition to their normal duties (Moosa 2020). On that same vein, OMs had to lead from the front as staff became fearful to assume their clinical roles of prevention and management of the COVID-19 pandemic as frontline workers. They had to deal with great confusion, uncertainty and difficult experience in the history of their nursing management. These managers were tasked with the difficult decision of distribution of limited resources, which posed a huge dilemma on them as facilities were still expected to perform optimally on the deliverables and also efficiently deal with the COVID-19 pandemic in the community (Greenberg 2020).

## Conceptual framework

**FIGURE 1:**
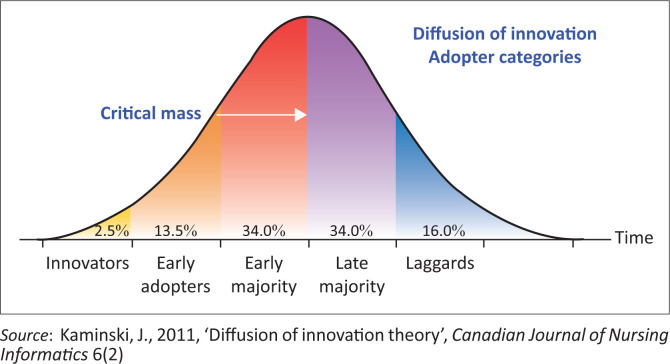
Diffusion of innovation adopter categories.

This study was guided by Rogers’ diffusion of innovation theory (Rogers 1962, 2003). Rogers defined this theory as a valuable change model for guiding a specific transformation or innovation, where it is modified and presented in ways that meet the needs across all categories of adopters, and it also stresses the importance of communication and peer networking within the adoption process. This author added that the diffusion of innovation outlines the process that happens when people implement a new idea, product, practice or philosophy. Categories of adopters as discussed by Rogers (1962, 2003) are (1) innovators, who are risk takers and peer educators; (2) early adopters are role models and respected by peers; (3) early majority – these managers avoid risks and only adopt processes from reliable trusted sources; (4) late majority – this group responds to peer pressure and are always doubters; and (5) laggards are comfortable to maintain the status quo. All these categories were observed within the OMs and nurses of Ekurhuleni health district during the COVID-19 pandemic. The findings of this study confirm that most of the OMs had to be the innovators and early adopters to support the early and late majority and the laggards. However, for these OMs to witness the success of this commitment, they had to be stretched beyond their capacity. Figure 1 presents a conceptual diagram of the categories of adopters as outlined by Rogers (1962, 2003).

## The aim and objectives of the study

The purpose and objective of this study were to explore and describe the PHC OMs’ experiences of new management dynamics in PHC facilities created by the COVID-19 pandemic in the Ekurhuleni health district.

## Research design and method

### Setting

This study was conducted in Gauteng, in the PHC facilities of Ekurhuleni district, East Region. This region has 28 facilities, operating five days a week from 07:30 to 16:30, with a few of them having extended hours on Saturdays, operating from 07:30 to 14:00. All these facilities offer comprehensive PHC services, including the management of the COVID-19 pandemic, such as screening, testing and vaccination. They have 25 OMs, one OM being responsible for one clinic. The other three OMs’ posts are currently vacant.

### Research design

The authors used a qualitative, explorative, descriptive and contextual research design to explore and describe facility managers’ experiences of new management dynamics in PHC services created by the COVID-19 pandemic in the Ekurhuleni health district. Research design is a comprehensive set of actions that is used to conduct a research study aimed at addressing the research purpose in greater depth (Bruce & Klopper 2017).

### Research method

A descriptive phenomenological research approach was used in this study to explore and describe the experiences of OMs on the phenomenon of interest (Christensen, Welch & Barr 2017; Holloway & Galvin 2017).

### Population and sampling

The population of this study was 78 OMs from Ekurhuleni regions. Purposive sampling was used to select the participants, and a total of 28 OMs became the sample of this study. These OMs had to meet the eligibility criteria to be able to assist the author with in-depth knowledge on the phenomenon, thus providing answers to the research question (Polit & Beck 2018). The eligibility criteria of the study were: OMs working in the Ekurhuleni East Region, with more than two years’ experience as an OM in the Ekurhuleni PHC facilities, and able to voluntarily give consent to be a participant in the study. Data saturation was reached by the seventh interview with no new data emerging from the participants, and two more interviews were conducted to confirm data (Polit & Beck 2018).

### Data collection

Data were collected using semistructured, in-depth, phenomenological interviews in order to receive valid and reliable data which would effectively explore and describe experiences of OMs on the new management dynamic in primary health care during the COVID-19 pandemic in Ekurhuleni health district. An informative session on the study was held with the participants in one of their operational meetings. Participants who showed interest were approached individually to elicit permission to participate. All participants who met the eligibility criteria and agreed to be part of the study were given information letters and consent forms for both interviews and audio recording. The interviews were conducted at the convenience of the participants, either at facilities’ boardrooms or in facility managers’ offices. The dates and times of the interviews were selected by the participants based on their availability. All venues were strategically chosen to ensure that there was adequate lighting, space for social distancing, comfortable chairs and table. Masks and sanitisers were readily available to ensure that COVID-19 protocols were adhered to, and water for drinking was available. The interviews were conducted between 01 March 2022 and 30 April 2022, by the author, who is one of the OMs in the same facilities, East Region.

The author had no personal relationship with any of the OMs, and preconceived ideas on the phenomenon and biasness were avoided by focusing on a central question and research purpose during data collection. The supervision sessions were held with the supervisors in between the data collection process to identify any bias during the interviews. The participants did not demonstrate any signs of having a problem being interviewed by one of their colleagues; instead, a majority of them were excited to share their experiences on the phenomenon. The duration of the interviews was about 45–60 min per interview. The participants were asked one central question: ‘What are the new management dynamics created by the COVID-19 pandemic in the PHC facility you are managing?’

### Data analysis

Data analysis was conducted using Giorgi’s descriptive thematic phenomenological data analysis to deduce meaning through the themes and subthemes (Polit & Beck 2018). Verbatim transcription, listening and re-listening to the audio recordings and attaching the field notes to the quotations of the participants assisted the author to avoid being biased during data analysis. The expertise of an experienced qualitative independent coder, who holds a master’s degree, was used to code and confirm the data. Discussion meetings with the independent coder, research supervisors and researcher were held to confirm and reach a consensus on the themes and subthemes that emerged from the data.

### Trustworthiness

To ensure trustworthiness in this study, the author used Lincoln and Guba’s (2013) framework of quality criteria, namely credibility, transferability, dependability and confirmability. Prolonged engagements with the participants in the field to extensively understand their context and triangulation of data were held to enhance validity and credibility of the data (LoBiondo-Wood & Haber 2018). Triangulation was used by collecting data using in-depth individual interviews and recording field notes; hence, bracketing was pre-empted through regular supervision feedback sessions during this process. To ensure the transferability of the study, the authors extensively discussed the research method and the context of the study. Dependability was maintained by holding regular corrections and feedback meetings with the supervisors of the study. Confirmability was ensured by back-and-forth meetings with the independent coder, researcher and supervisor to discuss the findings until consensus was reached. Peer review and audit were supported out on data.

### Ethical considerations

The following four ethical principles of Dhai and McQuoid-Mason (2020) were applied in this study: autonomy, beneficence, nonmaleficence and justice. Informed consent was obtained from participants who were willing to form part of the study. No direct benefits were promised to the participants; however, the findings of the study will be shared with the participants once the study results have been published by the university. No participant was forced to be part of the study and participants were given free will to withdraw anytime without any negative consequences. All participants were treated equally and with respect. Ethical clearance from University of Johannesburg Ethics Committee (reference number REC1264-2021) and from Ekurhuleni health district was granted to permit the researcher to conduct the study (Korstjens & Moser 2018).

## Results

In this study, data were collected from nine participants who were the OMs in the PHC facilities in Ekurhuleni, East Region. The minimum experience of the participants who were interviewed was three years to a maximum experience of 20 in the management role. Three participants were male and the rest of the six were female, with ages ranging from 38 to 61 years. All these managers had experience on PHC operational management before the COVID-19 pandemic, during and after the peaks of the pandemic. The Rogers diffusion of innovation theory (Rogers 1962, 2003), which underpinned this study, is apparent in the findings presented.

One central theme and three main themes surfaced from the findings of this study. The central theme was stretching of inadequate resources; subthemes included: (1) budgetary cuts and increasing demands of resources; (2) insufficient PPE, other general supplies and human resources; and (3) high staff turnover and increased client dissatisfaction. The field notes are presented in italics after the quotations from the participants.

### Stretching of inadequate resources

The participants (P) reported that they had to stretch the inadequate resources they have in their facilities in an attempt to respond to the demands of the COVID-19 pandemic. One participant went a step further and alluded that they had to soldier on, and this is in support of what Rogers’ theory reveals under innovators. This is confirmed by the following quotations from the participants:

‘And the other dynamics is that we had to work with inadequate resources in a demand increasing environment [*looking concerned*].’ (P9, 59 years, female)

‘Then when it comes to material resources [*yoh!*] We also struggled, [*yoh!*] We also struggled [*looking shocked*].’ (Participant 2, 61 years, female)

P6 added:

‘[*B*]ut since COVID-19 pandemic I had to constantly stretch myself to the extent that I was running on empty … yes, empty, but we had to soldier on.’ (42 years, male)

Operational managers in PHC are obligated to manage the facilities using the available resources (Jooste 2017). Nene, Ally and Nkosi (2020) argue that it is difficult to execute tasks effectively with limited resources. Brouer et al. (2016) went a step further and alluded that healthcare senior managers expected miracles of achieving the quality healthcare goals without proper supply of required resources. These authors also added that the healthcare demands are already escalating, and they require OMs to ensure control of resource utilisation, rate of use, flexibility, effective use of equipment, other general supplies and human resources.

The obligation of OMs to ensure that there are adequate resources in PHC facilities was a mission impossible during the COVID-19 pandemic peaks, as they had to stretch insufficient resources, and this included having to assume both management and clinical duties themselves, which include assessment, treatment and care (Shipton et al. 2016). Operational managers and other healthcare workers in these PHC settings were overloaded with work, and it negatively influenced their mental health. This is supported by Liu et al. (2020) when they elucidated that after the COVID-19 outbreak, healthcare workers were challenged as their working environment completely changed; they experienced exhaustion because of heavy workloads and insufficient PPE, the fear of becoming infected and infecting others, as well as feeling powerless to handle patients’ conditions and manage relationships in this stressful situation. Rogers (2003) annotated that innovators show up during difficult times and they develop creative strategies that will ease the pressure of stretching inadequate resources.

### Budgetary cuts and increasing demands on resources

The participants shared their experiences of new management dynamics, and these included budgetary cuts from the senior management and increasing demands on resources. The early adopters, early majority and late majority of Rogers’ (2003) diffusion of innovation theory are evidenced in one of the quotations from the participants. ‘Jumping from that, I want to talk about budget, we had a lot of constraints’ said P2 (42 years, male). P7 added that ‘[t]hat budget was now taken away, we had to adopt, some early and others late’ (38 years, female).

‘You are actually working even though you are managing.’ (P5, 52 years, female)

‘Meaning that they will be testing as well as seeing the patients in the consulting rooms which as well creates a very … very … big burden to them, because what happens is they do that and then the next two weeks they are not on duty because of exhaustion so it’s that and the resources.’ (P3, 43 years, male)

Budgetary cuts were the only method of survival for the under-resourced South African healthcare system during the COVID-19 pandemic outbreak (South African Department of Health, COVID-19 Risk Adjusted Strategy 2020) confirmed this by positing that the government responded to the COVID-19 pandemic with large-scale economic relief measures to support the most vulnerable South Africans, and measures will also build the capacity of the public health system to respond to the pandemic. Rogers (2003) pointed out that in the process of implementing transitional or transformational change effectively in the organisation, there have to be early adopters, early majority and late majority of change. The management of the PHC clinics of Ekurhuleni had to acknowledge that the OMs interpret change differently, and for effective change implementation, they had to be supported, irrespective of their category – whether they were early adopters, early or late majority.

Jooste (2017:205) and Nene (2022) revealed that it was also difficult for OMs to inspire their nurses, and they were all expected to do more than their capacity, and one cannot lead and follow at the same time. As the numbers of patients who were coming to PHC facilities for services were increasing in South Africa, no new staff members were employed to close the capacity (Brouer et al., 2016). It was scary enough that the COVID-19 pandemic variants were partially understood (WHO 2020), yet the frontline workers were not supported with enough resources. Zhang et al. (2020) posit that one question that nurses asked themselves is that are they expected to execute their obligations without necessary resources. According to Lai et al. (2020), the psychological well-being of OMs was gravely compromised during the COVID-19 pandemic, as a result of anxiety about becoming infected with the virus and long working hours contributing to their exhaustion.

### Insufficient personal protective equipment, other general supplies and human resources

The participants alluded that they were short-staffed and struggling with a shortage of resources, such as PPE, general supplies and personnel. The quotations confirm this statement. ‘We were always short staffed, worked overtime, but then like I said you have to do most of the things yourself’ said P5 (52 years, female).

Resources are the backbone of healthcare, and senior managers have to advocate for the availability of sufficient resources to ensure that quality healthcare is delivered (Jooste 2017). Operational managers have to do situational analysis to determine the amount of resources required in their facilities. In their study on resources during the COVID-19 pandemic, Thian (2015) concluded that nurses were overloaded with workload and time pressure that emanated from insufficient resource, and theywere frustrated by the lack of support from their managers during this time.

P2 and P7 added that:

‘From time to time, we were struggling with shortage of material resources, especially PPE’s and other general supplies, so there were limited general consumable medication and medical, it was not exactly explained why they were limited, because I believe all the money was diverted to PPE’s, the PPEs that didn’t come so we don’t know what happened but all the money was diverted to one area, in the context of saying “we are going to buy PPEs”.’ (P2, 61 years, female)

‘Personnel management was a problem because now and then the very same personnel were infected with the virus, and others were too scared and had to drag themselves to work.’ (P7, 38 years, female)

Nene et al. (2020) annotated that the South African healthcare system is troubled by insufficient resources, from equipment to material to human resources. During COVID-19, South Africa borrowed 500 billion rands from the World Bank, but corrupt politicians took advantage of this money and ensured that they would benefit from it (EWN 2020). During one of the national meetings, also known as South African family meeting, the South African government disclosed that tenders were awarded to the associates of the politicians without following a proper procurement process; consequently, some of the facilities ended up not getting the resources, such as PPE (South African Department of Health, COVID-19 Risk Adjusted Strategy 2020).

Zhang et al. (2020) concluded that infection control and discomfort caused by insufficient supply of PPE, general supplies and human resources during the COVID-19 pandemic became the most significant stress factors for the frontline nurses and OMs. The WHO (2020) shared the same sentiments and postulated that nurses were placed in a life-threatening position, as they had to care for the COVID-19 patients in an environment of increasing infection risk, without adequate resources. The WHO posited that some of the frontline workers were demotivated by this scary experience and had to drag themselves to work. This confirms what Rogers (2003) highlighted in the theory that during change, managers have to deal with laggards who are pulling themselves back during change. Jooste (2017) elucidate on laggards as change resisters who should be allowed to express their fears and feelings about the change that being introduced. Executive managers should come down to the operational level to address the concerns of those affected by change and to empower OMs for buy-in, as they are expected to be change catalysts.

### Compromised service delivery and increased client dissatisfaction

Most of the participants disclosed that they were experiencing a high influx of patients, and they could not provide quality care; hence, the complains of the clients escalated.

This is confirmed by the following quotations:

‘Yeah, eh … the normal flow of patients I said there’s an influx which there is no more quality care that was provided, we just wanted them to be out. Poor quality, service delivery, long waiting times.’ (P3, 59 years, female)

‘We really had to compromise, but remember if you do not have resources of treating the patient, you will not give the total quality patient care.’ (P7, 38 years, female)

Zhang et al. (2020) point out that the stress and excessive workloads in nursing staff during the COVID-19 pandemic directly affected their work engagement and quality of care provided to patients. The WHO (2020) lamented that, in low-resource settings, patients had to wait even more during the COVID-19 pandemic, and it is sad that some of them lost their lives while waiting to be attended to. Lipsitch and Phil (2020) annotated that it was more difficult to triage patients in PHC during COVID-19, because a majority of them required a secondary healthcare intervention, and yet the hospitals were also full. Muller (2020) and Nene (2021) elicit that the COVID-19 trying times required strong leaders who are resilient, agile and supportive and who can be hands-on to ensure to improve service delivery.

Another participant said:

‘But now it’s five or ten complaints within a day as a result of waiting time, and shortage of staff due to infections, so I will find myself needing to write more items on relation to complaints. Everything is a mess, I cannot even plan properly, I just can’t.’ (P6, 44 years, male)

Basit (2017) reveals that it is devastating for the OMs to receive complains from the patients, because this act questions the quality of their leadership. With such an influx of patients and insufficient resources, it was not a surprise that the complaints of patients increased during this pandemic (Lipsitch & Phil 2020). Bao et al. (2020) and the National Health Commission (2020) concluded that what frontline nurses required during the outbreak of COVID-19 was support with adequate resources, psychological support, logistics support, timely and open communication.

## Discussion

### Limitations

This study was limited and focused on the experiences of only OMs on the new management dynamic brought by the COVID-19 pandemic in Ekurhuleni health district, East Region only. This limited the scope and overview of the study, as other provinces of the country and the other categories of staff working in the PHC facilities where the study was conducted did not form part of the study, namely medical practitioners, registered nurses, administrators, cleaning personnel, etc.

### Implications of the study

The findings of this study can be implemented in PHC facilities to effectively deal with future pandemics of such a nature, and to protect OMs and nurses during such times.

### Recommendations

It is recommended from this study that the South African government improve the supply of resources during pandemics to ensure that the healthcare system is responsive and to create a healthy working environment for OMs and frontline nurses. Senior managers of healthcare and OMs have to be trained to effectively deal with pandemics of this nature. Clear guidelines and protocols should be developed for management of global pandemics and national disasters, with specific role specification for each category, avoiding abrupt and uncoordinated responses in case of a pandemic as experienced during the COVID-19 pandemic.

## Conclusion

This study revealed that OMs were over-stretched and overwhelmed by the management of PHC facilities because of extended duties that came with the COVID-19 pandemic. Crisis management and limited resources placed a good deal of stress and anxiety on the well-being of the OMs. Human resource management created undesirable strain as workers were physically and psychologically strained by the pandemic and being in the forefront of fighting against this deadly virus. The COVID-19 pandemic decreased and compromised the quality of nursing care and patient safety, with compromised practical competencies and capabilities of PHC operations leading to poor performance of PHC facilities on expected deliverables and increased client dissatisfaction.
